# Validation of the Arabic version of the Cohen perceived stress scale (PSS-10) among pregnant and postpartum women

**DOI:** 10.1186/1471-244X-10-111

**Published:** 2010-12-15

**Authors:** Monique Chaaya, Hibah Osman, Georges Naassan, Ziyad Mahfoud

**Affiliations:** 1 Professor, Department of Epidemiology and Population Health, Faculty of Health Sciences, American University of Beirut, Beirut, Lebanon; 2 MPH Assistant Professor, Department of Health Promotion and Community Health, Faculty of Health Sciences, American University of Beirut, Beirut, Lebanon; 3 Department of Health Promotion and Community Health, Faculty of Health Sciences, American University of Beirut, Beirut, Lebanon; 4 Associate Professor, Department of Public Health, Weill Cornell Medical College, Doha, Qatar

## Abstract

**Background:**

This study was conducted to evaluate the validity of the Arabic translation of the Cohen Perceived Stress Scale (PSS-10) in pregnant and postpartum women.

**Methods:**

A sample of 268 women participated. These included 113 women in their third trimester of pregnancy, 97 in the postpartum period and 58 healthy female university students. GHQ-12 and EPDS were also administered to the participants. Internal consistency reliability, assessed using Cronbach's α, was 0.74.

**Results:**

PSS-10 significantly correlated with both EPDS and GHQ12 (ρ = 0.58 and ρ = 0.48 respectively), and significantly increased with higher scores on stressful life events. PSS-10 scores were higher among university students who also recorded higher stressful life events scores.

**Conclusion:**

The Arabic translated version of the PSS-10 showed reasonably adequate psychometric properties.

## Background

Addressing stress during pregnancy and the postpartum period is important as these periods are physically, psychologically and socially distinct periods in a women's lifetime during which mothers experience concerns about the health of their child, their own health, changes in their bodies and the subsequent effect on changes in their marital relationship. Additionally, worries regarding economic insecurity, breastfeeding, and bonding with the infant can exacerbate the stress often experienced in this period [1,2]. First-time mothers have the added stressors of adapting to their new role as mothers and the insecurities associated with their ability to nurture an infant for the first time [3-5]. These stressors have a significant impact on the mother's psychological well being such as prenatal and postpartum depression, especially when stressors are perceived as stressful [6]. Perceived stress is a person's appraisal of certain life events as potentially threatening. This perception is reached in light of the person's ability to cope with such events [7,8]. Therefore people evaluate potentially stressful life events differently. The ability to accurately measure perceived stress is essential in order to treat and evaluate the effectiveness of interventions and treatments.

There are few validated tools for the measurement of stress during pregnancy or during the postpartum period [9-11]. One commonly used scale is the 10-item Cohen Perceived Stress Scale (PSS-10). The PSS-10 has been used to research stress among different population groups including healthy university students, drug addicts, elderly populations, as well as pregnant and postpartum women. The Cohen PSS was originally developed in 1983 as a 14-item Likert type questionnaire in order to measure one's own perception and appraisal of life events as stressful [12]. A shorter version emerged when the psychometric properties of the PSS-14 scale were assessed using data from phone interviews with 2387 male and female US residents from all ages, ethnicity and household income [13]. Four questions were dropped from the PSS-14 when it was found that they did not load on either of the two factors obtained using exploratory factor analysis for the PSS-14. The PSS-10 was found to have adequate reliability and validity and a slightly higher internal reliability than PSS-14 (Alpha coefficient of 0.78 vs. 0.75). Exploratory factor analysis of PSS-10 uncovered the same two- factor structure as PSS-14. The first factor included questions reflecting negative feelings (being upset, angry, or nervous) and inability to handle stress while the second factor included questions expressing positive emotions and ability to act in stressful situations. This has been confirmed in more than one study that examined psychometrics of self-administered PSS-10 among different populations [14,15].

The 10 items in the scale inquire about feelings and thoughts that tap the degree to which respondents find their current life situation unpredictable, uncontrollable and stressful. Respondents indicate how often in the past month they have felt or thought a certain way on a 5-point Likert scale (0 = never, 1 = almost never, 2 = sometimes, 3 = fairly often, 4 = very often). The higher the score the higher the perceived stress is. The scale correlates with different psychosocial measures specifically depression, anxiety, and perception of poor health as well as with decreased satisfaction with self, job and life in general [13,14].

The PSS-10 has been translated to different languages including Arabic, Spanish, Turkish, Mexican Swedish, Greek, Bulgarian, Chinese, Thai, Japanese, Persian, and Hungarian [16]. However, not all translated versions of PSS-10 have been validated. Hamdan-Mansour and Dawani [17] used an Arabic version of PSS-10 to study stress among university students in Jordan. They reported an internal consistency of 0.68. Using the pilot data of a study investigating stress and health among working Jordanian women, Hattar-Pollara and Dawani [18] reported a Cronbach alpha of the Arabic translated version of 0.86. The authors of the latter study recommended further testing of the PSS-10 to establish its construct validity and ensure that the scale is culturally sensitive.

Although PSS-10 has been used in many studies to measure stress among pregnant and postpartum women [19,20], none of these studies were designed for validation purposes among these populations. However, some of these studies reported a good level of reliability with internal consistency of the PSS-10 scale ranging from 0.71 to 0.83 [19,21,22].

The aim of this study was to translate the PSS-10 to classical Arabic and examine its psychometric properties among pregnant and postpartum women. Specifically, the objectives of the study were to assess the reliability of the Arabic PSS-10, its concurrent validity with similar validated scales used in pregnancy and the postpartum periods, and its construct validity by examining its ability to detect meaningful variance between specific groups of the population. A reliable and validated Arabic tool for measuring stress among women in the postpartum period, such as the Arabic version of the PSS-10 is essential for assessing the impact of interventions aimed at decreasing stress levels in the postpartum period among Arabic speaking women in different countries.

## Methods

### Participants

Overall 268 women participated in the study. These included 113 women in their third trimester (starting week 28 of pregnancy), and 97 women in the postpartum period (within 6 months after delivery). Moreover, 58 healthy female university students; who were neither pregnant nor mothers; were recruited to act as controls. Pregnant and postpartum mothers were recruited through the clinics of two obstetricians and one paediatrician during prenatal, postpartum or well baby visits. The three clinics chosen provided care to patients from different socio economic backgrounds. Consecutive pregnant and postpartum women attending the chosen clinics were approached and all consented to the study (100% response rate). Female university students were selected using quota sampling from the six academic units at the American University of Beirut, with 10 from each unit. Only two students out of 60 approached refused to participate in the study (97% response rate). Data collection was done over a two- month period.

### Instrument

The first step of the validation process was the translation of the original English version of PSS-10 to classical Arabic by a professional translator. Classical Arabic was chosen to make the tool useful for all Arabic speaking countries, as "spoken" Arabic can be very different between countries. After translation, the scale was then reviewed by a bilingual psychiatrist for appropriateness of language. The reviewed version of the translated PSS-10 was then back translated by the psychiatrist into English and compared to the original one in order to check for consistency. Both translator and psychiatrist were not familiar with the scale. Discrepancies were corrected accordingly. The obtained Arabic PSS-10 was piloted on a small group of mothers (n = 10) to ensure that all the terms employed were understandable and to modify any ambiguity. Finally, a sample of bilingual female university students (n = 10) was asked to complete both the English and the Arabic versions of the PSS-10 [Additional file 1] consecutively on the same day. The mean time between the two administrations was 5 minutes. The Spearman correlation coefficient (rho) between the English and the Arabic versions was 0.71. Those 20 women were not included in the final sample used in this study.

Moreover, the validity of the PSS-10 was assessed by concurrently administering the Arabic versions of the General health Questionnaire (GHQ-12) to all participants and the Edinburgh Postpartum Depression Scale (EPDS) to pregnant and postpartum women. The GHQ-12 is a short version of the 60-item GHQ developed by Goldberg [23] to screen for psychological disorders in primary care and community settings. Its use in research and in the clinical settings was previously established in a study conducted by the World Health Organization in 1995 [24]. The Arabic GHQ-12 has a sensitivity of 83%, a specificity of 80%, and a total discriminatory power of 86% [25]. The EPDS is a 10-item screening instrument developed to identify postnatal depression in health care settings and it has been used extensively for research [24,26]. The Arabic version of EPDS was validated by Ghubash and Abou-Saleh [25]. Using a cut-off score of 10 the sensitivity and specificity of the Arabic EPDS were 91% and 84% respectively and the internal reliability using Cronbach α was 0.84. Both EPDS and GHQ are validated rating scales to screen for psychological distress and depression among women in the prenatal and postpartum periods [27,28]. PSS-10 has been reported to correlate positively with GHQ-12 (r = 0.61 with n = 508) [15] and EPDS (r = 0.52 with n = 130) [29]. We expected a positive association between the three scales PSS-10, GHQ-12 and EPDS.

In addition to comparison with the GHQ-12 and the EPDS, the validity of the PSS-10 was evaluated by comparing the PSS scores among the 3 groups studied while adjusting for variables unbalanced between the three groups and potentially affecting the PSS-10 scores. We expected that pregnant and postpartum women would be more stressed than healthy university students.

### Administration

After informed consent was obtained, the selected participants answered a structured questionnaire that included the three Arabic scales: PSS-10, GHQ-12, and EPDS (for pregnant and postpartum women only). Basic demographic information (such as age and level of education) was solicited. As an indicator of socio economic status (SES), women were asked whether their income was sufficient. Moreover, the women were provided with a list of 7 negative life events and were asked if they experienced any in the past year and how much they were affected (no impact, little impact, moderate impact and severe impact). These events were: divorce/separation in the family, problems at work/university, death of a family member or a close friend, illness of a family member, financial problems, personal problems (that required effort to deal with), and health problems related to pregnancy/delivery. Correlating PSS scores with current stressful life events scores provided an additional means for assessing validity.

To check for reliability, a test-retest procedure was performed where 60 participants; 20 from each group, were chosen at random and asked to consent to a retest after one week. Of those, 41 women (20 students, 17 pregnant and 4 postpartum women) accepted (68% response rate). Postpartum women were reluctant to come back to the clinic for a retest after one week.

The questionnaire was self-administered; however, in case selected participants were unable to read or had problems reading the scales, the recruiters in each clinic read the scales to them and noted their responses. The study was approved by the Institutional review Board at the American University of Beirut.

### Data Analysis

Descriptive statistics (means with standard deviations or frequency distributions) on age, education, work status, and perceived adequacy of income were calculated for each sample and for the combined samples. Comparisons of such demographic variables between the three samples were done using one-way ANOVA for comparing age, the chi-squared test for comparing work status, and Fisher exact test for comparing education and perceived adequacy of income [Table 1].

**Table 1:** Distribution of Demographic Variables among the Samples

	Total (N = 268)	University Student (n = 58)	Pregnant Woman (n = 113)	Postpartum Woman (n = 97)	p-value
**Age **mean ± sd	27.6 ± 5.5	22.5 ± 3.1	28.4 ± 5.7	29.7 ± 4.7	<.01
**Education**					<.01*
Primary	26 (10%)	0 (0.0%)	17 (15%)	9 (10%)	
Secondary	66 (25%)	0 (0.0%)	34 (30%)	32 (34%)	
University	173 (65%)	58 (100%)	62 (55%)	53 (56%)	
**Work Status**					.02
Works	96 (36%)	25 (45%)	30 (27%)	41 (43%)	
Does not work	168 (64%)	31 (55%)	83 (73%)	54 (57%)	
**Perceived Income sufficiency**					<.01
Barely sufficient	13 (5%)	4 (7%)	4 (4%)	5 (5%)	
Almost	134 (51%)	10 (18%)	71 (65%)	53 (55%)	
Most of the times	65 (25%)	24 (42%)	20 (18%)	21 (22%)	
More than enough	52 (20%)	19 (33%)	15 (14%)	18 (19%)	

* University students were significantly more educated than both pregnant women and postpartum women. However, there was no difference (p-value = 0.47) between pregnant women and postpartum women.

The internal consistency reliability and the test-retest reliability of the Arabic PSS-10 were assessed using Cronbach's alpha coefficient and Spearman's correlation coefficient (rho) respectively. Internal consistency was evaluated for the combined sample as well as for each of the three women groups (student, pregnant, and postpartum) [Table 2]. The test-retest reliability was performed for the combined group of all women who accepted to do the retest. Subgroup reliability measures were computed for the student group and the combined pregnant and postpartum participants since only 4 pregnant women did the retest.

**Table 2:** Arabic PSS-10 Internal Consistency Reliability and Its Correlation with other Scales

Group	Crobach's Alpha for PSS-10	Spearman's Rho for correlation with GHQ12	Spearman's Rho for correlation with EPDS	Spearman's Rho for correlation with life events score
Student	.74	.56*	NA	.37*
Pregnant	.75	.56*	.45*	.27*
Postpartum	.71	.67*	.56*	.29*
All Three	.74	.59*	.49*	.30*

* all entries were significant at the .05 level

Exploratory factor analysis was performed using principal components with varimax rotation [Table 3]. Mean PSS-10 and GHQ-12 scores were compared between the three groups using the one-way ANOVA. Post-hoc comparisons were done using Bonferroni's method. Mean EPDS scores were compared between the pregnant women group and the postpartum women group using the independent t-test [Table 4].

**Table 3:** Results of the Factor Analysis: Components and factor loadings

PSS-10	Component 1	Component 2
1. In the last month, how often have you been upset because of something that happened unexpectedly?	.57	-.32
2. In the last month, how often have you felt that you were unable to control the important things in your life?	.63	-.20
3. In the last month, how often have you felt nervous and "stressed"?	.76	-.18
4. In the last month, how often have you felt confident about your ability to handle your personal problems?	.07	.58
5. In the last month, how often have you felt that things were going your way?	-.23	.60
6. In the last month, how often have you found that you could not cope with all the things that you had to do?	.57	.13
7. In the last month, how often have you been able to control irritations in your life?	.01	.75
8. In the last month, how often have you felt that you were on top of things?	-.17	.70
9. In the last month, how often have you been angered because of things that were outside your control?	.69	.05
10. In the last month, how often have you felt difficulties were piling up so high that you could not overcome them?	.77	-.08

**Table 4:** Means and Standard Deviations of the scores for all 4-scales

	Student	Pregnant	Postpartum	p-value	Adjusted p-value
PSS-10	20.3 (4.8)^A^	18.0 (5.7)^B^	18.3 (4.8)^B^	.02*	0.29^†^
GHQ-12	14.0 (5.1)	12.1 (5.0)	13.1 (4.8)	.06	
EPDS	------	9.0 (5.5)	8.3 (4.8)	.33	
Total score of events	3.5 (3.3)^A^	2.7(2.9)^B^	1.9(2.6)^C^	<.01*	

* Different superscript letters indicate significant difference between the means for the unadjusted pairwise comparisons using Bonferroni's method

†adjusted for age, total score of events, education, work status, and perceived income adequacy

Questions about stressful life events were coded from 0 (event did not occur or event occurred and the participant said that it had no impact on her) to 3 (event occurred and participant said it had a severe impact on her) with increasing values indicating increased impact of the event on a person's life. A stressful life event score was computed by adding the scores of all the questions about stressful life events. This event score was compared among the three groups using the Kruskal-Wallis test. Post-hoc comparisons were made using Bonferroni's method. Its correlation with the PSS-10 score was computed using Spearman's correlation coefficient. Finally, a multivariable analysis of covariance (ANCOVA) regression model was fit with PSS-10 score as the response variable and group as the independent variable (using two indicator variables as group is a nominal variable) while adjusting for the following covariates: age, educational level, stressful life event score, work status, and perceived income adequacy.

## Results

The mean age of the 268 participants was 27.6 years with a standard deviation of 5.5 years. The majority (64%) did not work while a high proportion (51%) perceived that their income was almost adequate for their needs. In both the postpartum group and the pregnant group, the majority (55% and 56%) of women were university educated. University students were significantly younger, more educated, and a higher proportion (75%) of them considered their income sufficient (defined as being adequate for their needs "most of the time" or "more than enough") as compared to the two other groups (32% for pregnant women and 41% for women in the postpartum period) [Table 1]. Apart from work status, demographic variables were not significantly different between pregnant and postpartum samples. Except for two, all participants were able to fill in the questionnaires by themselves. Excluding those two women from the data analysis did not change the significance of any of the results; hence the two women were kept in the final sample used for this analysis.

For the overall sample, Cronbach's alpha for assessing the internal consistency reliability of the Arabic PSS-10 was 0.74. It ranged from 0.71 for postpartum women to 0.75 for pregnant women [Table 2]. Factor analysis showed that the scale is composed of two components with eigen values of 3.1 and 1.6 and accounting for 47.3% of the variance (data not shown). Component one consisted of questions 1, 2, 3, 6, 9, and 10 and component 2 consisted of the questions 4, 5, 7, and 8 [Table 3]. The test-retest reliability of the Arabic PSS-10, was moderately high with Spearman's correlation coefficient of 0.74. Reliability was higher (0.79) among students as compared to the other two groups (0.63). Time to retesting was one week for university students and varied between 2 to 3 weeks for participants from the other two groups.

As for evaluation of validity, the Arabic PSS-10 exhibited significant positive correlations with both GHQ-12 and EPDS [Table 2]. Spearman's Rho values were higher for correlations with GHQ12, when considering both the sub-samples and the total samples, and indicated moderate association. EPDS correlated better with PSS-10 in postpartum women than in pregnant women. There was also a significant positive correlation (Spearman's Rho = .30) between PSS-10 and the score of stressful life events. That association was highest among students as compared to the other two groups [Table 2].

Examining the sensitivity of PSS-10 to different population groups, the results showed that PSS-10 scores were significantly higher among the students as compared to both pregnant women and postpartum women [Table 4]. No significant difference was observed between pregnant and postpartum women. Similar trend was observed for GHQ-12, however differences were not statistically significant (p = 0.06). As for stressful life events scores, both university students and pregnant women scored significantly higher than postpartum women [Table 4]. In particular, for each stressful life event, mean PSS-10 scores for those who reported severe impact were significantly higher than all other categories. Moreover, for each event, a higher proportion of university students reported experiencing a severe impact than the two other groups (data not shown). On the other hand, there was no significant difference in the mean EPDS score for the pregnant and postpartum groups.

Finally, differences in PSS-10 scores among the three groups became non significant (p = 0.29) after adjusting for age, education, perceived adequacy of income, work status, and total event score using the multivariable ANCOVA regression model.

## Discussion

This is the first study designed to evaluate the reliability and validity of the Arabic PSS-10 scale. This Arabic translation of the PSS-10 was found to have reasonably adequate psychometric properties. Among university students, the reliability coefficient was similar to that found by Cohen and Williamson [13]. For pregnant and postpartum women the estimates fell within the range (.71-.82) of previously reported values in other studies on similar groups [19,22,30]. The overall test retest reliability of .74 was acceptable and comparable to that obtained for the Spanish version of PSS-10 [31]. However, reliability was higher among the students as compared to the other two groups possibly due to the difference in time to retest. Results of the factor analysis were similar to those obtained from studies in Turkey [15] and the US [12] where the same two factors were detected. Örücü and Demir [15] had described these two factors as "perceived helplessness" and "perceived self efficacy". As hypothesized, the Arabic PSS-10 had positive associations with two previously validated scales: GHQ-12 and EPDS. There was also a significant positive correlation between PSS-10 and life events scale but that correlation was of a lower magnitude. This might be due to differences in the way people cope with life events, thus people with stressful life events and good coping strategies might have a higher score on life event scale however a lower score on PSS-10. Further, studies on relating stress to life events are recommended taking into account the coping strategies of people.

The mean PSS-10 score of the sub-sample of students in this study was similar to what is reported in Arab female university students (22.7 ± 7) by Hamdan-Mansour and Dawani [17]. Contrary to our expectations, scores of university students on PSS-10 were higher than those of pregnant or postpartum women. One possible reason is that the students' sample unexpectedly experienced significantly higher stressful life events than the other two groups. Further studies for assessing the sensitivity of PSS-10 to distinguish between different stress levels among postpartum and pregnant women need to be pursued.

### Study Limitations

This study was conducted among specific groups of women, the use of PSS-10 in studies about women of different groups or men should also try to address internal consistency and factor structure in those populations. Moreover, women were recruited from three clinics and one private university, and therefore our sample may not be representative of all pregnant, postpartum, and university women. This study was not able to establish the ability of PSS-10 to distinguish between different stress levels and further investigation in that direction is recommended.

One other limitation of the study was that retesting among pregnant and postpartum women was done after two to three weeks as compared to one week for students. Events might have happened thereby affecting the scores in the second interview and thus lowering the reliability.

## Conclusions

Stress is a risk factor for several chronic diseases including hypertension, diabetes, and coronary artery disease. The ability to measure stress reliably would be useful to further characterize the link between stress and health. More importantly it would help evaluate interventions that may decrease stress levels.

PSS-10 can be used to estimate perceived stress. It is short and can be easily administered to women coming to seek medical help.

The availability of a valid PSS-10 scale in different languages allows for comparisons across studies from different countries and cultures that ultimately help in understanding reactions to stress and its determinants. The translation of PSS-10 into classical Arabic makes this a useful tool for researchers conducting studies that address stress in a variety of Arabic-speaking communities.

## Competing interests

The authors declare that they have no competing interests.

## Authors' contributions

MC, HO, and GN, contributed to conceptualization, and write up. ZM and MC, contributed to data collection, analysis and write up. All authors have read and approved the manuscript being submitted.

## Pre-publication history

The pre-publication history for this paper can be accessed here:

http://www.biomedcentral.com/1471-244X/10/111/prepub

## Supplementary Material

Additional file 1 **The Arabic Version of PSS-10**. This is the final version of the Arabic version of the PSS-10 that was administered to the three women groups. Click here for file
