# Supplementary material for: Validation of the Arabic version of the Cohen perceived stress scale (PSS-10) among pregnant and postpartum women
Source: BMC Psychiatry. 2010 Dec 15;10:111. doi: 10.1186/1471-244X-10-111 (PMC3016315; doi:10.1186/1471-244X-10-111)
Supplement: Additional file 1 — The Arabic Version of PSS-10. This is the final version of the Arabic version of the PSS-10 that was administered to the three women groups. [file 1471-244X-10-111-S1.DOC]

**. مقياس التوتر النفسي**

| **تدور الأسئلة في هذا المقياس حول أحاسيسك وأفكارك** خلال الشهر الذي مضى**. الرجاء الاجابة عن كل حالة ووضع إشارة كم من الوقت شعرت او فكرت بهذه الطريقة:** | | | |
| --- | --- | --- | --- |
| **بتاتاً** | 0 | **خلال الشهر الذي مضى، كم من الوقت شعرت بالاضطراب نتيجة حصول حادثة غير متوقعة؟** | Stress 1 |
| **شبه بتاتاً** | 1 |  |  |
| **أحياناً** | 2 |  |  |
| **غالباً** | 3 |  |  |
| **غالباً جداً** | 4 |  |  |
| **بتاتاً** | 0 | **خلال الشهر الذي مضى، كم من الوقت شعرت بعدم قدرتك على التحكم بالأمور المهمة في حياتك؟** | Stress 2 |
| **شبه بتاتاً** | 1 |  |  |
| **أحياناً** | 2 |  |  |
| **غالباً** | 3 |  |  |
| **غالباً جداً** | 4 |  |  |
| **بتاتاً** | 0 | **خلال الشهر الذي مضى، كم من الوقت شعرت بالتوتر و"الضغط النفسي"؟** | Stress 3 |
| **شبه بتاتاً** | 1 |  |  |
| **أحياناً** | 2 |  |  |
| **غالباً** | 3 |  |  |
| **غالباً جداً** | 4 |  |  |
| **بتاتاً** | 0 | **خلال الشهر الذي مضى، كم من الوقت شعرت بالثقة حيال قدرتك على التعامل مع مشاكلك الشخصية؟** | Stress 4 |
| **شبه بتاتاً** | 1 |  |  |
| **أحياناً** | 2 |  |  |
| **غالباً** | 3 |  |  |
| **غالباً جداً** | 4 |  |  |
| **بتاتاً** | 0 | **خلال الشهر الذي مضى، كم من الوقت شعرت أن الأمور تجري حسب مشيئتك؟** | Stress 5 |
| **شبه بتاتاً** | 1 |  |  |
| **أحياناً** | 2 |  |  |
| **غالباً** | 3 |  |  |
| **غالباً جداً** | 4 |  |  |
| **بتاتاً** | 0 | **خلال الشهر الذي مضى، كم من الوقت شعرت بعدم قدرتك على التكيف مع جميع الأمور التي عليك فعلها؟** | Stress 6 |
| **شبه بتاتاً** | 1 |  |  |
| **أحياناً** | 2 |  |  |
| **غالباً** | 3 |  |  |
| **غالباً جداً** | 4 |  |  |
| **بتاتاً** | 0 | **خلال الشهر الذي مضى، كم من الوقت شعرت انك قادر على التحكم بمصادر الازعاج في حياتك؟** | Stress 7 |
| **شبه بتاتاً** | 1 |  |  |
| **أحياناً** | 2 |  |  |
| **غالباً** | 3 |  |  |
| **غالباً جداً** | 4 |  |  |
| **بتاتاً** | 0 | **خلال الشهر الذي مضى، كم من الوقت شعرت انك تتحكم بجميع الأمور؟** | Stress 8 |
| **شبه بتاتاً** | 1 |  |  |
| **أحياناً** | 2 |  |  |
| **غالباً** | 3 |  |  |
| **غالباً جداً** | 4 |  |  |
| **بتاتاً** | 0 | **خلال الشهر الذي مضى، كم من الوقت شعرت بالغضب بسبب أمور خارجة عن سيطرتك؟** | Stress 9 |
| **شبه بتاتاً** | 1 |  |  |
| **أحياناً** | 2 |  |  |
| **غالباً** | 3 |  |  |
| **غالباً جداً** | 4 |  |  |
| **بتاتاً** | 0 | **خلال الشهر الذي مضى، كم من الوقت شعرت ان المصاعب تتراكم لدرجة لا يمكنك التحكم بها؟** | Stress 10 |
| **شبه بتاتاً** | 1 |  |  |
| **أحياناً** | 2 |  |  |
| **غالباً** | 3 |  |  |
| **غالباً جداً** | 4 |  |  |
